# LEARNING FROM ELDERS: WORKING WITH INDIGENOUS INTERGENERATIONAL MENTORS TO ADDRESS COMMUNITY NEEDS

**DOI:** 10.1093/geroni/igac059.238

**Published:** 2022-12-20

**Authors:** Karen Kopera-Frye, Karen John, Robynn Frank

**Affiliations:** New Mexico State University, Las Cruces, New Mexico, United States; New Mexico State University, Las Cruces, New Mexico, United States; New Mexico State University, Las Cruces, New Mexico, United States

## Abstract

Tribal Critical Race Theory (Brayboy, 2005) supports the use of decolonizing methodologies such as Community-Based Participatory Research when collaborating with Indigenous communities. This paper highlights the underlying processes in working with a Dine community on an intergenerational health project. COVID had culturally disruptive effects, e.g., social isolation, on New Mexico’s Dine community. This project describes what can be best thought of as Community-Based Participatory Advocacy (Kopera-Frye, John, & Frank, 2021). Navajo students interviewed 13 area chapter elders on how COVID has impacted the community, particularly with Indigenous Ways of Knowing (IWOK). Thematic analysis indicated themes of loss, stress and social isolation, and health worker effects. Resilience was indicated in response to positive outcomes from COVID including a collaboration and coming together of community. Open dialogue workshops are ongoing to facilitate community healing from COVID. The results highlight the critical necessity of starting with the community elders and using decolonizing methodologies.

